# Outcome after resection of Adrenocortical Carcinoma liver metastases: a retrospective study

**DOI:** 10.1186/s12885-017-3506-z

**Published:** 2017-08-04

**Authors:** Johannes Baur, Tjark-Ole Büntemeyer, Felix Megerle, Timo Deutschbein, Christine Spitzweg, Marcus Quinkler, Peter Nawroth, Matthias Kroiss, Christoph-Thomas Germer, Martin Fassnacht, Ulrich Steger, on behalf of the German Adrenocortical Carcinoma Study Group

**Affiliations:** 1 Department of General, Visceral, Vascular and Pediatric Surgery, University Hospital, University of Wuerzburg, Wuerzburg, Germany; 2 Department of Internal Medicine I, Division of Endocrinology and Diabetes, University Hospital, University of Wuerzburg, Wuerzburg, Germany; 3 Department of Internal Medicine IV - Campus Grosshadern University Hospital of Munich, Ludwig-Maximilians-University Munich, Munich, Germany; 4 Endocrinology in Charlottenburg, Berlin, Germany; 5 Department of Medicine I and Clinical Chemistry, University Hospital, University of Heidelberg, Heidelberg, Germany; 6 Comprehensive Cancer Center Mainfranken, University of Wuerzburg, Wuerzburg, Germany

**Keywords:** Adrenocortical carcinoma, Liver resection, Retrospective study, Prognosis, Survival analysis

## Abstract

**Background:**

Metastatic Adrenocortical Carcinoma (ACC) is a rare malignancy with a poor 5-year-survival rate (<15%). A surgical approach is recommended in selected patients if complete resection of distant metastasis can be achieved. To date there are only limited data on the outcome after surgical resection of hepatic metastases of ACC.

**Methods:**

A retrospective analysis of the German Adrenocortical Carcinoma Registry was conducted. Patients with liver metastases of ACC but without extrahepatic metastases or incomplete tumour resection were included.

**Results:**

Seventy-seven patients fulfilled these criteria. Forty-three patients underwent resection of liver metastases of ACC. Complete tumour resection (R0) could be achieved in 30 (69.8%). Median overall survival after liver resection was 76.1 months in comparison to 10.1 months in the 34 remaining patients with unresected liver metastases (*p* < 0.001). However, disease free survival after liver resection was only 9.1 months. Neither resection status (R0/R1) nor extent of liver resection were significant predictive factors for overall survival. Patients with a time interval to the first metastasis/recurrence (TTFR) of greater than 12 months or solitary liver metastases showed significantly prolonged survival.

**Conclusions:**

Liver resection in the case of ACC liver metastases can achieve long term survival with a median overall survival of more than 5 years, but disease free survival is short despite metastasectomy. Time to recurrence and single versus multiple metastases are predictive factors for the outcome.

## Background

Adrenocortical Carcinoma (ACC) is a rare malignancy with an annual incidence of 0.7-2.0 cases per million population [1] with a poor prognosis [2–4]. Treatment strongly depends on tumour staging classification suggested by the European Network for Study of Adrenal Tumours (ENSAT) [5]. Here, primary tumours are classified into four groups (I-IV). ENSAT stage I and II include T1 and T2 primary ACC tumours without lymphatic involvement or distant metastases. Locally advanced tumours or ACC with lymphatic metastases but without distant spread are classified as ENSAT stage III. In these tumour stages, complete resection of primary tumour as well as regional lymphadenectomy with adjuvant mitotane therapy is recommended even if multivisceral resection is required [2, 6]. Here, disease-specific 5-year-survival rates of 82%, 61% and 13% for stage I, II and III are reported [5].

Metastatic (ENSAT stage IV) and recurrent ACC disease exhibit poor 5-survival rates of less than 15% [7]. Nevertheless, if complete resection of distant metastases or recurrent tumour can be achieved, a surgical approach is recommended [7, 8] In a series of 154 patients analysed after first recurrence, complete resection of recurrent disease led to a median overall survival of 88 months, when R0 status was achieved, compared to 11 months in cases in whom surgery was not possible [9]. Similar results were seen in a recent series from France with 59 patients [10]. In the case of metastatic disease of ACC, the liver is one of the most involved organs beside the lung. In contrast to patients with isolated liver metastases (LM) of colorectal origin, in which surgical resection is recommended due to a 5-year overall survival rate of about 42% after first liver resection [11], there is as yet only limited data about the value of liver resection for non-colorectal, non-neuroendocrine metastases of other infrequent primary tumours such as ACC [12].

In the present study, we report on a retrospective analysis with a high case load using the German Adrenocortical Carcinoma Registry and focusing on a more homogeneous group of patients with resected and non-resected isolated liver metastases without extrahepatic manifestation of ACC origin to proof the value of liver resection for ACC LM.

## Methods

### Data source

The German Adrenocortical Carcinoma Registry, which was established in 2003 [13], was used for analysis. Patients having diagnosis of liver metastases from Adrenocortical carcinoma until the end of 2015 were involved in this study, and data of 1031 patients could be included. Clinical data was collected by trained medical personnel using structured evaluation forms containing comprehensive information on diagnostic procedures, surgical outcomes, and follow-up. The German Adrenocortical Carcinoma Registry was approved by the ethics committee at the University of Wuerzburg, and patients gave written informed consent.

### Patient selection

**Fig. 1:**
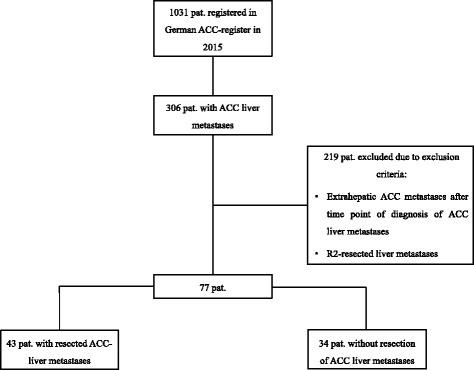
Flow chart of the ACC registry patients and the selection process for patients with liver metastases and no extrahepatic manifestation

From the registry, a total of 306 patients with ACC liver metastases (29.7%) were identified. Of those, 219 patients were excluded, while 77 patients, who were at least 18 years of age, met the inclusion criteria of ACC liver metastases without extrahepatic metastatic disease at the time of diagnosis and without incomplete resection (R2 including debulking) (Fig.1).

Of the patients included in the study, 43 patients had undergone radical liver resection under curative intention between 1985 and 2015. The histologic diagnosis for each patient was made by the local pathologist. The remaining 34 patients with isolated ACC liver metastases had not undergone liver resection. Reasons for non-surgical treatment were rarely recorded. Reasons for this could be missing interdisciplinary tumour conferences in former times, the multicentre setting or a limited performance status as a reason for patients not being eligible for liver resection. To consider possible inhomogeneities characteristics like patient age at diagnosis of metastases, sex, side of primary tumour, diameter of the primary tumour, hormone status of the primary, the appearance of liver metastases (simultaneous or secondary), number of liver metastases, the extent of the surgical procedure and adjuvant therapies like mitotane were obtained from the registry and in the most cases of the selected patients directly by review the medical records. All patients were followed up on a regular basis (usually every 3 months). Therefore, a complete analysis of overall and disease free survival was possible.

### Statistical analysis

Statistical analysis between the groups was carried out by using χ2 test for categorical endpoints. A t-test was used for continuous endpoints. Survival analysis was performed using the Kaplan-Meier method and log rank test. A univariate analysis for relevant prognostic factors of overall and disease-free survival was performed using the Cox proportional hazards model. All factors that showed a trend in overall and disease-free survival in the univariate analysis (*p* < 0.1) were further investigated in a multivariate analysis. *P*-values <0.05 were considered statistically significant.

## Results

### Patient characteristics

Until 2015, 77 patients with liver metastases of ACC but without extrahepatic manifestation at the time of first appearance of the liver metastases were identified from the registry. Mean age at diagnosis of metastases was 49.8 years. There was a significant difference in the mean age at diagnosis between the group that underwent liver resection (46.0 years) and the nonsurgical group (54.7 years) (Table 1). The primary tumour was equally distributed on both sides. However, liver resection was performed more frequently, when the primary was localized on the right side (60.5%). The number of patients with a primary tumour size of >10 cm was significantly higher in the non-resection group (82.4%) compared to the surgical group (55.8%). In both groups, around 50% of the carcinomas were hormone-secreting. However, there was a high percentage of patients with unknown hormone status in the non-resection group (32.4%). 69.8% of resected liver metastases occurred secondary or metachronous, whereas 58.8% occurred simultaneous in the non-resection group. There was also a significantly higher percentage of patients with solitary metastases in the resection group. Systemic therapies after the diagnosis of liver metastases like mitotane or other chemotherapies were more often applied in the non-resection group than after resection. Mitotane was used in 64.7% of patients without liver resection, whereas it was administered after resection in only 48.8%. Other drugs like etoposide, doxorubicin and cisplatin were applied in 47.1% and 18.1% respectively (Table 1).

**Table 1:** Basic patients’ characteristics

	overall		Liver resection		no Liver resection		*p*-value
	n	(%)	n	(%)	n	(%)	
Patiens							
overall	77		43		34		
female	56	(72.7%)	30	(69.8%)	26	(76.5%)	0.512
Mean age at first diagnosis [y]	49.5		45.5		54.5		0.016
Mean age at diagnosis of liver metastases [y]	49.8		46.0		54.7		0.017
Primary tumour							
Localisation							
right gland	38	(49.4%)	26	(60.5%)	12	(35.3%)	0.061
left gland	38	(49.4%)	17	(39.5%)	21	(61.8%)	
both glands	1	(1.3%)	0	(0.0%)	1	(2.9%)	
Diameter							
> 10 cm	52	(67.5%)	24	(55.8%)	28	(82.4%)	< 0.001
≦ 10 cm	17	(22.1%)	16	(37.2%)	1	(2.9%)	
unknown	5	(6.5%)	0	(0.0%)	5	(14.7%)	
Hormon status at presentation							
functional	38	(49.4%)	21	(48.8%)	17	(50.0%)	< 0.001
non-functional	21	(27.3%)	15	(34.9%)	6	(17.6%)	
unknown	11	(14.3%)	0	(0.0%)	11	(32.4%)	
Liver metastases							
Simultaneous liver metastases	33	(42.9%)	13	(30.2%)	20	(58.8%)	0.012
Secondary liver metastases	44	(57.1%)	30	(69.8%)	14	(41.2%)	
Time between first diagnosis and liver metastases [m]	43.9		60.5		8.4		0.002
Number							
1	35	(45.5%)	26	(60.5%)	9	(26.5%)	< 0.001
2-5	12	(15.6%)	7	(16.3%)	5	(14.7%)	
> 5	20	(26.0%)	0	(0.0%)	20	(58.8%)	
unknown	10	(13.0%)	10	(23.3%)	0	(0.0%)	
Systemic therapies after diagnosis of liver metastases							
Mitotane	43	(55.8%)	21	(48.8%)	22	(64.7%)	0.164
Chemotherapy	24	(31.2%)	8	(18.6%)	16	(47.1%)	0.007

### Liver resection for ACC metastases

In the group of surgical metastasectomy, minor resections of 1 or 2 segments were most frequently performed regardless of a secondary or simultaneous approach (Table 2). Histologically confirmed complete tumour removal (R0 status) was achieved in around 70% of the cases, with a better performance status when the procedure was done secondarily. Repeated liver resection was carry out in one third of the cases.

**Table 2:** Liver Resection: Procedural Details

overall	secondary resection		simultaneous resection	
Extend of first liver resection				
1 segment	15 (34.9%)	9	(30.0%)	6 (46.2%)
2 segments	13 (30.2%)	8	(26.7%)	5 (38.5%)
mutltiple segments	1 (2.3%)	1	(3.3%)	0 (0.0%)
hemihepatectomy	7 (16.3%)	6	(20.0%)	1 (7.7%)
extended hemihepatectomy	3 (7.0%)	2	(6.7%)	1 (7.7%)
atypical resection	2 (4.7%)	2	(6.7%)	0 (0.0%)
unknown	2 (4.7%)	2	(6.7%)	0 (0.0%)
Resection Status of first liver resection				
R0	30 (69.8%)	23	(76.7%)	7 (53.8%)
R1	8 (18.6%)	5	(16.7%)	3 (23.1%)
Rx	5 (11.6%)	2	(6.7%)	3 (23.1%)
Systemic Therapies after first liver Resection^a^				
Mitotane	21 (48.8%)	17	(56.7%)	4 (30.8%)
Chemotherapy	8 (18.6%)	3	(10.0%)	5 (38.5%)

^a^Application within 3 Months after Liver reseection

### Overall and disease free survival

**Fig. 2:**
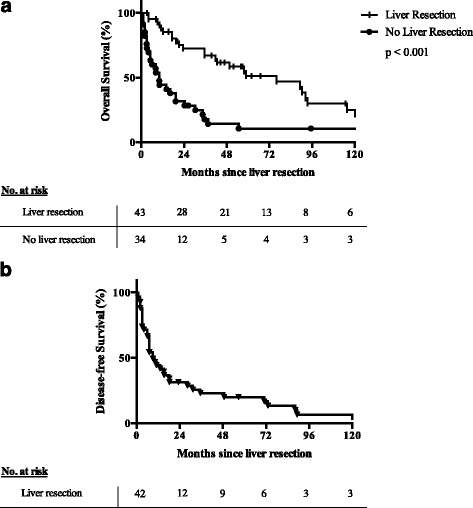
**a** Overall survival (OS) after liver resection for ACC liver metastases in comparison to the non-resected group. **b** Disease-free survival after liver resection

After liver resection of metastases of adrenocortical origin, the median overall survival was 76.1 months in comparison to 10.1 months in the 34 remaining patients with unresected liver metastases (*p* < 0.001) The 5-year survival rate was 51.3% after surgical therapy vs. 10.7% in the control group without resection. Nevertheless, the disease-free survival after liver resection was only 11.1 months with a 5-year disease free survival rate of 20.1%. (Fig.2).

### Predictive factors

**Fig. 3:**
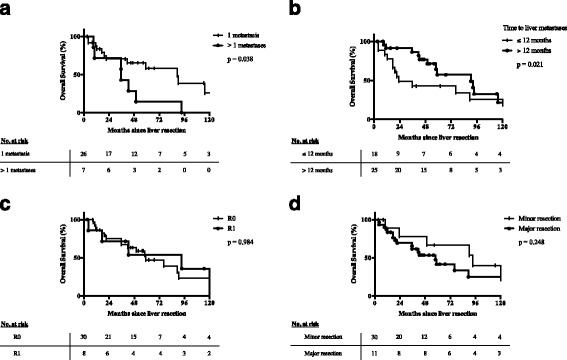
Overall survival after liver resection of ACC liver metastases for patients with (**a**) 1 or more metastases, (**b**) time to recurrence of less or greater than 12 month, (**c**) R0 and R1 resection and (**d**) major (>2 segments) or minor liver resection

When comparing the different characteristics between patients in the resection group, only patients with a time interval to the first metastasis/recurrence (TTFR) of greater than 12 months or solitary liver metastases showed significantly prolong survival (Fig. 3, Table 3). In the multivariate analysis the hazard ratio continued to be in the same range (2.455 and 2.876 respectively), but this was not anymore significant. Neither resection status (R0/R1) nor the extent of liver resection (major/minor) were significant predictive factors for overall survival. Sex, age, side of primary tumour, size of primary tumour, hormone status or additive mitotane therapy all had no significant influence on overall outcome. Application of chemotherapies other than mitotane within 3 months after liver resection was associated with even poorer survival (Table 3). This reflects, however, likely a selection bias, because 50% of these patients experienced recurrence of ACC within 3 months after liver resection and therefore had a poor prognosis on survival.

**Table 3:** Predictive factors on overall survival after liver resection

			Univariate analysis			Multivariate analysis		
	n	median survival [mo]	HR	95% CI	*p*-value	HR	95% CI	*p*-value
Sex								
female	30	76.1	1					
male	13	58.9	1.066	0.444 - 2.563	0.886			
Age at first liver resection (yr)								
≤ 50	13	93.3	1					
> 50	30	41.6	2.225	0.830 - 5.964	0.112			
Occurence of liver metastases								
secondary	30	89.3	1					
simultaneous	13	23.3	1.485	0.673 - 3.278	0.327			
Major liver resection								
no	30	57.8	1					
yes	11	93.3	0.580	0.226 - 1.488	0.257			
Number of liver metastases								
1	26	89.3	1			1		
> 1	7	35.5	2.620	1.008 - 6.808	0.048	2.455	0.888 - 6.786	0.083
Pathologic margin								
R0	30	58.9						
R1	8	93.3	1.037	0.372 - 2.889	0.945			
Rx	5	92.3	1.127	0.401 - 3.167	0.821			
Side of primary tumour								
right	26	76.1	1					
left	17	89.3	0.772	0.349 - 1.707	0.522			
Size of primary tumour (cm)								
≤ 10	16	92.3	1					
> 10	24	41.6	2.059	0.855 - 4.954	0.107			
Secreting primary tumour								
no	15	90.3	1					
yes	21	42.6	1.624	0.665 - 3.962	0.287			
Chemotherapy after liver resection								
no	35	90.3	1			1		
yes	8	17.3	12.176	3.765 - 39.373	< 0.001	3.453	0.774 - 15.410	0.104
Mitotane after liver resection								
no	22	90.3	1					
yes	21	57.8	1.010	0.446 - 2.288	0.981			
Time to liver metastases [mo]								
> 12	25	90.3	1			1		
≤ 12	18	23.3	1.983	0.916 - 4.292	0.082	2.876	0.770 - 10.7431	0.116

Significant predictive factors on the disease-free survival were number of liver metastases, size of primary tumour and chemotherapy after liver resection (Table 4).

**Table 4:** Predictive factors on disease-free survival after liver resection

			Univariate analysis			Multivariate analysis		
	n	median survival [mo]	HR	95% CI	*p*-value	HR	95% CI	*p*-value
Sex								
female	30	10.2	1					
male	13	7.1	1.062	0.528 - 2.136	0.866			
Age at first liver resection (yr)								
≤ 50	13	35.5	1					
> 50	30	7.1	1.552	0.759 - 3.173	0.229			
Occurence of liver metastases								
secondary	30	9.1	1					
simultaneous	13	7.1	1.309	0.640 - 2.677	0.461			
Major liver resection								
no	30	7.1	1					
yes	11	31.4	0.558	0.256	1.216	0.142		
Number of liver metastases								
1	26	11.2	1			1		
> 1	7	4.1	2.490	1.028 - 6.034	0.043	3.747	1.292 - 10.865	0.015
Pathologic margin								
R0	30	10.2	1					
R1	8	3.1	1.859	0.827 - 4.175	0.133			
Rx	5	73.1	0.809	0.276 - 2.377	0.700			
Side of primary tumour								
right	26	7.1	1					
left	17	15.2	0.710	0.367 - 1.374	0.309			
Size of primary tumour (cm)								
≤ 10	16	18.2	1			1		
> 10	24	7.1	2.034	0.985 - 4.198	0.055	2.764	1.062 - 7.192	0.037
Secreting primary tumour								
no	15	13.2	1					
yes	21	9.1	1.008	0.490 - 2.071	0.983			
Chemotherapy after liver resection								
no	35	15.2	1			1		
yes	8	3.1	2.844	1.193 - 6.777	0.018	2.568	0.936 - 7.043	0.067
Mitotane after liver resection								
no	22	7.1	1					
yes	21	10.2	0.994	0.517 - 1.912	0.985			
Time to liver metastases [mo]								
> 12	25	18.3	1					
≤ 12	18	6.1	1.754	0.895 - 3.436	0.102			

### Recurrence after liver resection

Recurrence after the first liver resection of ACC metastases was frequent (88.4%) regardless of the timepoint (synchronous or metachronous) of metastatic occurrence. In 57.9% of cases, recurrence occurred isolated in the liver, followed by lung (28.9%), abdominal cavity (18.4%) and bone (7.9%). Multifocal recurrence occurred in only 10.5% of all resected cases (4/43). There were only minor differences in the distribution pattern of recurrence between synchronous and metachronous liver metastases (Table 5).

**Table 5:** Recurrence after first Liver resection

	overall	secondary resection	simultaneous resection
Recurrence after first liver resection	38 (88.4%)	27 (90.0%)	11 (84.6%)
Median Overall Survival [mo]	57.8	58.9	19.3
Median disease-free survival [mo]	11.1	10.2	11.2
Localisation of Recurrene			
Liver	22 (57.9%)	15 (55.6%)	7 (63.6%)
Abdomen	7 (18.4%)	6 (22.2%)	1 (9.1%)
Lung	11 (28.9%)	7 (25.9%)	4 (36.4%)
Bone	3 (7.9%)	2 (7.4%)	1 (9.1%)
multiple Localisations	4 (10.5%)	2 (7.4%)	2 (18.2%)

## Discussion

In this retrospective study, we analysed the benefit of surgical resection of isolated ACC liver metastases based on the German ACC registry. Although the registry contained a substantial number of patients with liver metastases of adrenocortical origin, accounting for 29.7% (306/1031) of registered patients, only 77 of these patients presented with isolated liver metastases and either received surgical resection or systemic therapies alone.

Irrespective of the number of metastases, our analyses demonstrate a very high 5-year survival rate of 51.3% in the 43 liver resected ACC patients. Our data are consistent with previous studies showing similar outcomes after resection of hepatic metastases of ACC origin. In a cohort study of resected non-colorectal and non-neuroendocrine liver metastases of 28 patients with ACC, Adam et al. reported a 5-year overall survival of 66% and a median survival of 63 months after liver resection [14]. Based on these data, ACC patients were ranked as the most favourable group for liver resection after non-colorectal and non-neuroendocrine hepatic metastases by these authors. Based on 19 liver patients with metastatic ACC, Ripley et al. reported a 5 year overall survival of 29% after resection, and a 5 year overall survival of 29% patients (*n* = 5) that underwent local hepatic thermoablation (RFA) [15]. Another case report and review of the literature about thermoablation for ACC LM suggested that RFA should be considered as therapy alternative only for patients in whom resection is contraindicated, due to the increased risk of local recurrence in highly vascularized ACC metastases after ablation [16]. Weitz et al. analysed 15 patients with ACC and liver metastases with a 40 months cancer specific survival after resection [17]. A subsequent report from the same institution included 28 patients with ACC LM with a disease-free survival of 7 month and a 5 year survival of 39% [18]. The latter studies did not exclude patients with extrahepatic tumour manifestation. Thus, the overall outcomes in these studies are reasonably poor compared data obtained from our selected resection group which excluded extrahepatic metastases. Additional data on patients presenting ACC LM suitable for resection but that were never resected is rare or not available in the literature.

Predictive factors to allocate the patient to radical surgery in case of recurrence, seems to be a time to first recurrence (TTFR) of greater than 12 months and a solitary occurrence of liver metastasis. Whereas Erdogan et al. also identified R0 resection as a predictive factor for prolonged overall survival in advanced ACC [9], our data did not show a significant difference between R0 and R1 liver resections. A similar effect was shown for colorectal liver metastases, where patients with R1 hepatic resection achieve similar overall survival rates as R0 resected patients, despite a higher recurrence rate [19]. This fact could be unique in the case of metastasectomy in the liver. However, we have to acknowledge that the number of our study is too small to prove this hypothesis.

Importantly, however, the results of surgical treatment could not be achieved by nonsurgical therapies alone, such as mitotane or other chemotherapies. For mitotane monotherapy in advanced ACC, distinct data about overall survival in patients without resection is difficult to obtain. Some reports indicate tumour response rates of 13% to 33% [20]. However, as recently described in the FIRM-ACT trial, even the combination of mitotane with etoposide, doxorubicin and cisplatin (EDP) resulted in an overall survival of only 14.8 month in advanced ACC [21]. In addition, the results of other medical approaches are also of limited or no efficacy [22–27].

Mitotane was shown to have a beneficial effect as adjuvant treatment after radical resection of primary ACC [28]. In our study, we did not observe a significant effect of mitotane in the adjuvant/additive setting after resection of ACC liver metastases. Despite the low number of patients, this result might suggest that adjuvant therapy after metastasectomy of ACC by mitotane or chemotherapy does not appear to be very beneficial. Similarly, in a study of 27 patients with synchronously metastatic ACC Dy et al. observed no apparent impact of chemotherapy after metastasectomy on overall survival. Nevertheless, neoadjuvant chemotherapy in the form of mitotane with or without other chemotherapeutic agents like etoposide, doxorubicin and cisplatin was shown to improve overall survival after resection in 8 patients [29]. Based on these data, neoadjuvant treatment prior to metastasectomy should be considered as a possible pathway in further drafts of trials for stage IV ACC.

In the group of liver resection, the site of primary ACC was more often on the right side (60%), whereas an equal distribution of the primary ACC to both sides was observed in all 77 patients with isolated liver metastases. Therefore, direct infiltration of the primary tumour into the liver on the right side simulating a solitary metastasis cannot be ruled out for all patients. However, our data suggest that the side of the primary had no significant impact on the overall survival after liver resection (see Table 3). So, the matter of direct tumour infiltration can be neglected.

Reliable information about surgical complications in our study collective could not be obtained from the register. However, by looking at the length of hospital stay and a low in-house mortality it can be assumed that the complication rate of ACC liver metastasectomy is similar to resection of other secondary liver tumours like colorectal liver metastases.

Limitations of the study are its retrospective design and the fact that even after excluding 219 patients with liver metastases and extrahepatic manifestation or with incomplete resection, there are still major differences in the basic characteristics between the two groups of resected and non-resected patients, including median age, diameter of the primary tumour or number of metastases that complicate objective comparison of overall survival.

Yet, due to the rareness of ACC and the fact that a large ACC register was the base for this analysis, larger patient collectives than provided in this study with resected isolated ACC liver metastases and an additional corresponding homogenous control group might be hard to achieve.

## Conclusions

Liver resection can achieve long term survival in stage IV ACC with a 5-year survival rate of 51.3% in this study. However, disease-free survival seems to be short as the median disease-free survival is only 9.1 months despite radical metastasectomy. The main predictive factors for improved outcome appear to be the interval between primary tumour manifestation and hepatic recurrence as well as occurrence of a single versus multiple metastases within the liver.

## Abbreviations

ACC: Adrenocortical carcinoma
ENSAT: European Network for Study of Adrenal Tumours
LM: Liver metastases
OS: Overall survival
RFA: Radiofrequency ablation
TTFR: Time interval to first metastasis/recurrence
